# Metabotropic Glutamate Receptor 8 Is Regulated by miR-33a-5p and Functions as an Oncogene in Breast Cancer

**DOI:** 10.1155/2021/8002087

**Published:** 2021-12-14

**Authors:** Chunxu Zhang, Shuang Xie, Shouxin Yuan, Yuanhao Zhang, Yunhu Bai, Lijia Chu, Zuyin Wu, Ninghui Guo, Quanhui Wang, Jixin Zhang

**Affiliations:** ^1^Department of General Surgery, PLA No. 988 Hospital of Joint Logistics Support Force, Zhengzhou, Henan 450042, China; ^2^Department of General Surgery, PLA No. 984 Hospital of Joint Logistics Support Force, Beijing 100000, China

## Abstract

It has been reported that glutamate metabotropic receptor 8 (GRM8) is closely implicated in the progression of human neuroblastoma, lung cancer, and glioma, but its role in breast cancer remains unknown. Thus, the present study was performed to uncover it. Immunohistochemistry, real-time PCR (RT-PCR), and western blotting experiments were performed to test GRM8 expression levels in tissues and cells. Cell functions were assessed by Cell Count Kit 8 (CCK-8), flow cytometry, wound healing, transwell chambers, and *in vivo* xenotransplantation experiments. The relationship between miR-33a-5p and GRM8 was evaluated by luciferase gene reporter and western blotting assay. The results showed that GRM8 expression was increased in breast cancer tissues and cells, which was closely associated with lower overall survival rate. Ectopic expression of GRM8 significantly enhanced cell growth, migration, and invasion and tumorigenesis and repressed cell apoptosis. In addition, GRM8 was under the negative regulation of miR-33a-5p, which was downregulated in breast cancer tissues and served as a tumor suppressor. Moreover, overexpression of GRM8 abrogated the inhibitive role of miR-33a-5p played in breast cancer. Collectively, this study reveals that GRM8 functions as an oncogene in breast cancer and is regulated by miR-33a-5p.

## 1. Introduction

With 2.26 million new cases estimated in 2020, female breast cancer has now become the most common cancer over the world [1]. Also, it is the leading cause of cancer-related death in women worldwide mainly due to its high metastasis [2, 3]. Although many genes were discovered to play important roles in the progression of breast cancer [4], lots of genes' functions still need to be clarified.

Glutamate metabotropic receptors (GRMs) belong to the family of G-protein coupled receptors, which can be activated by glutamate and then activate the phospholipase C/protein kinase C/calcium, phosphatidylinositol 3-kinase/Akt/mammalian target of rapamycin (PI3K/AKT/mTOR), and mitogen-activated protein kinase (MAPK) pathways [5]. The GRM family contains eight members, GRM1, 2, 3, 4, 5, 6, 7, and 8, with GRM1 and GRM5 in group I, GRM2 and GRM3 in group II, and GRM4, 6, 7, and 8 in group III [6]. Several studies have reported that GRM8 is closely implicated in several kinds of cancers. For instance, Zhang et al. [7] demonstrated that GRM8 significantly enhanced the survival of squamous cell lung carcinoma cells through inhibiting cAMP pathway and activating MAPK pathway, suggesting that GRM8 serves as an oncogene in squamous cell lung carcinoma. On the contrary, Jantas et al. [8] reported that GRM8 overexpression significantly inhibited cell proliferation and enhanced cell chemosensitivity in human neuroblastoma (SH-SY5Y) and glioma (LN229, U87-MG, and LN18) cells, suggesting that GRM8 functions as a tumor-suppressive gene in human neuroblastoma and glioma. These findings indicate different roles of GRM8 plays in various types of cancers.

MicroRNAs (miRNAs) are a class of endogenous and noncoding RNAs and come from pri-miRNAs and pre-miRNAs with 21–24 nt in length [9, 10]. Evidence demonstrates that miRNAs can function as posttranscriptional regulators to induce translational repression of the target genes [11]. Many miRNAs have been reported to play important roles in the progression of breast cancer, such as miR-204-5p [12], miR-574 [13], miR-19b [14], miR-199a-5p [15], and miR-200a [16]. miR-33a-5p expression has been demonstrated to be declined in triple-negative breast cancer (TNBC) cells as compared to non-TNBC cells, and miR-33a-5p significantly increases TNBC cell sensitivity to doxorubicin (Dox) and attenuates epithelial-mesenchymal transition (EMT) [17]. In addition, miR-33a-5p could significantly inhibit the glycolysis of TNBC cells [18]. These results indicate that miR-33a-5p is implicated in the progression of breast cancer. Based on the bioinformatics results, we found that miR-33a-5p is a predicted upstream regulator of GRM8. However, whether miR-33a-5p targets GRM8 and then involves in the progression of breast cancer remains unclear.

In the present study, we aimed to explore GRM8 effect on the progression of breast cancer, as well as to reveal the relationship between miR-33a-5p and GRM8, and the role of miR-33a/GRM8 axis in breast cancer progression.

## 2. Materials and Methods

### 2.1. Tissue Samples

Eighty breast cancer tissues and the paired paracarcinoma normal tissues (≥3 cm from the cancer tissues) were obtained from 80 cases of breast cancer patients between 2008 and 2010 in our hospital prior to chemoradiotherapy. All tissue samples were immediately stored at −80°C after surgery. All patients signed the informed consents before this study and gave consent to have their data published.

### 2.2. Immunohistochemistry (IHC) Experiment

IHC staining was performed to evaluate the expression of GRM8 protein in 80 human breast cancer tissues and the adjacent noncancerous tissues. In brief, paraffin sections were cut into 4 *μ*m thick, followed by being deparaffinized with xylene and rehydrated with ethanol. Then, EDTA (ethylene diamine tetraacetic acid) and 3% H_2_O_2_ were used for antigenic retrieval and removing of endogenous peroxidase activity. Subsequently, the sections were immersed in 5% goat serum for 1 h at room temperature, followed by incubation with anti-GRM8 antibody (1 : 150 dilution, No. ab53094, Abcam, MA, USA) at 4°C overnight. Then, the sections were probed with the corresponding secondary antibody after rushing 3 times with PBS (phosphate buffer saline), followed by incubation with chromogen 3,3′-diaminobenzidine tetrachloride (DAB) (Serva, Heidelberg, Germany). Cell nucleus was stained with Harris hematoxylin solution (1 : 5000 dilution).

### 2.3. Cell Lines and Culture Conditions

Human breast cancer cell lines, including HCC1937, Bcap-37, MDA-MB-231, MCF7, and SK-BR-3, and one normal breast epithelial cell line (Hs 578Bst) were purchased from American Type Culture Collection (ATCC). Hs 578Bst cells were maintained in Hybri-Care Medium (ATCC), supplemented with 30 ng/ml EGF and 10% fetal bovine serum (FBS; Gibco, MA, USA). HCC1937 and Bcap-37 cells were grown in medium containing 90% RPMI (Roswell Park Memorial Institute)-1640 medium (Gibco) and 10% FBS. MDA-MB-231 and MCF7 cells were maintained in DMEM-H medium (Gibco) with 10% FBS. SK-BR-3 cells were incubated in 90% McCoy's 5a medium (Gibco) and 10% FBS.

### 2.4. Lentiviral Obtainment and Transfection

Short hairpin RNAs (shRNAs) used to downregulate GRM8 expression (sh-GRM8), the lentiviral overexpression vector (Vector-GRM8), the mimics and inhibitors targeting miR-33a-5p, and the corresponding negative controls (NC) were obtained from GenePharma Co., Ltd. (Shanghai, China). For lentivirus infection, 1 × 10^5^ breast cancer cells were placed in each well of the 6-well plates and allowed to adhere at 37°C overnight, followed by lentiviral infection with the help of polybrene. To establish the stable transfection cells, the transfected cells were incubated with puromycin (5 *μ*g/mL) and/or G418 (100 *μ*g/mL) for two weeks.

### 2.5. RNA Isolation and Real-Time PCR (RT-PCR)

Total RNA was isolated from cells and tissues with the help of TRIzol reagent (Invitrogen, Carlsbad, CA, USA). Then, the complementary DNA (cDNA) was synthesized using the PrimeScript™ Reverse Transcription kit (Takara, Tokyo, Japan) and TaqMan MicroRNA Reverse Transcription Kit (Takara) for mRNA and miRNA detection, respectively. The RT-PCR assay was carried out using the SYBR-Green Master Mix kit (Takara) on an ABI 7500 System (Applied Biosystems, MA, USA). GAPDH and U6 serve as internal reference for mRNA and miRNA, respectively. Primers were obtained from Shanghai Sangon Biotech (Shanghai, China) and listed in Table 1.

### 2.6. Western Blotting Assay

Protein was extracted from tissues and cells using RIPA lysis buffer (Roche, Shanghai, China), supplemented with 1% protease inhibitor (Solarbio, Beijing, China) according to the manufactory's instructions. After centrifugation at 4°C for 30 min, the Bicinchoninic Acid Protein Assay kit (Thermo Fisher Scientific) was used for protein quantification. Then, the protein samples were loaded to 10% SDS-polyacrylamide gel and separated by electrophoresis and transferred into the polyvinylidene difluoride membranes (PVDF; Millipore, Billerica, MA, USA). After incubation with 5% nonfat milk for 1 h at room temperature, the membranes were submitted to antibody incubation, including primary anti-GRM8 antibody (No. ab53094, Abcam) and the secondary antibody. Protein signaling was enhanced with ECL (enhanced chemiluminescence) reagent (Millipore) and detected with ProfiBlot-48 (Tecan, Switzerland) after being washed three times with PBS. The gray-scale value analysis was carried out using ImageJ software (National Institutes of Health).

### 2.7. Dual-Luciferase Reporter Assay

The Luciferase Reporter vectors, including Luc-GRM8-3′ UTR (untranslated regions) wild-type (WT) and mutant-type (MUT), were constructed by GenePharma. SK-BR-3 and HCC1937 cells were seeded into 96-well plates at a 60% confluence and incubated at 37°C overnight, followed by cotransfection with mimic-33a-5p or mimic-NC, and WT or MUT. The firefly and Renilla luciferase fluorescence activities were measured by a Dual-Luciferase Reporter system (Promega, Madison, WI, USA) after 48 hours of cell transfection.

### 2.8. Cell Proliferation Assessment by CCK-8 Assay

Cells with different vector transfections were inoculated into 96-well plates at a density of 3 × 10^3^/100 *μ*L. Cell counting kit-8 (CCK-8) assay was performed after 1, 2, 3, 4, or 5 days after inoculation. In detail, 10 *μ*L of CCK-8 reagent was added into each well, and the cells were cultured at 37°C for 3 h. The OD (optical density) value was measured at 450 nm.

### 2.9. Cell Apoptosis Detection

Cell apoptosis was assessed using an Annexin V-FITC/PI (propidium iodide) apoptosis detection kit (BD Biosciences, San Jose, CA, USA). Cells were collected and rushed with PBS for once time and were resuspended with binding buffer. Then, the cells were incubated with Annexin V-FITC and PI solution for 15 min at room temperature. Cell apoptosis was detected using a flow cytometer FACSCalibur system (BD Biosciences) and analyzed using FlowJo 7.6 software.

### 2.10. Wound Healing Assay

For wound healing assays, SK-BR-3 and HCC1937 cells were cultured in a 6-well plate until they reached 100% confluence. Then, the wounds were made with 20 *μ*l tip, and the medium was replaced with FBS-free medium. After 24 h of scratching, the scratch wound was observed and recorded by an inverted microscope with 6 randomly selected fields.

### 2.11. Transwell Chamber Assay

SK-BR-3 and HCC1937 cells with different transfections were seeded at a concentration of 1 × 10^5^ cells per well in FBS-free medium. After 48 h of the incubation, cells in the upper chamber of the transwell chamber (8 *μ*m; BD Biosciences) were removed with cotton swabs, while the invaded cells in the lower chamber were stained with 0.1% crystal violet (Solarbio). Cell invasion ability was assessed by counting whole invaded cell numbers under an optical microscopy at 100× magnification.

### 2.12. *In Vivo* Xenotransplantation Experiments

The BALB/c female nude mice at 6 weeks old were purchased from Beijing Vital River Laboratory Animal Technology Co., Ltd. (Beijing, China) and used for the xenotransplantation experiment. All mice were housed in a specific pathogen-free animal facility with free access to water and food. The animal experiment was approved by the Ethics Committee of PLA No. 988 Hospital of Joint Logistics Support Force.

Mice were subcutaneously injected in the armpit area with stably transfected SK-BR-3 (5 × 10^6^). After 28 days of the injections, mice were euthanized and the tumours were removed and weighed.

### 2.13. Statistical Analysis

Each measurement was performed in quintuplicate. All data are expressed as the means ± standard deviations (SD). The statistical analysis was carried out by SPSS23.0 with Student's *t*-test or One-Way Analysis of Variance (ANOVA). All of the reported *P* values are two-sided, and the *P* value <0.05 was considered statistically significant.

## 3. Results

### 3.1. GRM8 Is Overexpressed in Breast Cancer Tissues and Cells

**Figure 1:**
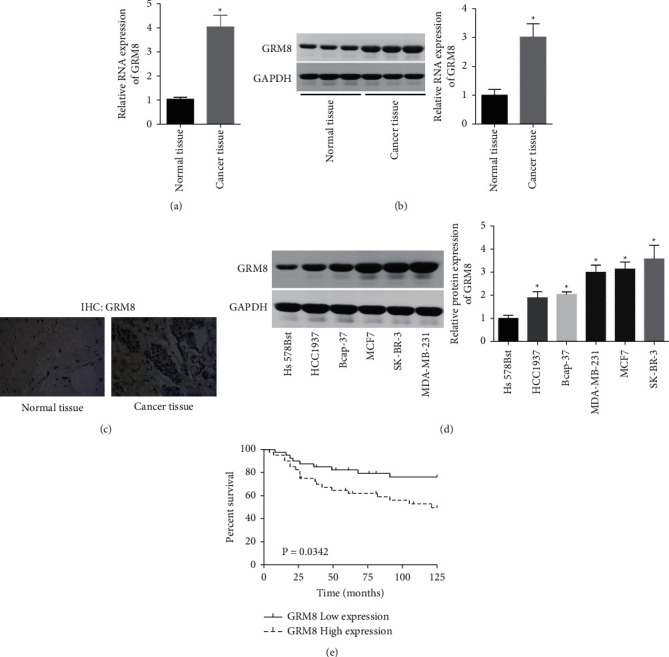
Breast cancer tissues and cells showed a high expression pattern of GRM8. (a) The mRNA levels of GRM8 in 80 paired breast cancer tissues and normal tissues were determined by RT-PCR. (b) The expressions of GRM8 protein in 3 paired breast cancer tissues and normal tissues were detected by western blotting. (c) IHC technology was performed to assess GRM8 expression patterns in breast cancer tissues and normal tissues. (d) GRM8 expression in different cell lines was determined by western blotting assay. (e) The clinical value of GRM8 in predicting the overall survival rate of breast cancer patients was assessed by the survivorship curve (^*∗*^*P* < 0.05).

To explore GRM8 role in the progression of breast cancer, we first assessed its expression profiles in breast cancer tissues and cells through RT-PCR, western blotting, and/or IHC technology. Compared with the adjacent normal tissues, the expression of GRM8 was significantly elevated in breast cancer tissues at mRNA (Figure 1(a)) and protein levels (Figures 1(b) and 1(c)). Similarly, GRM8 showed an increased expression pattern in breast cancer cell lines, including HCC1937, Bcap-37, MDA-MB-231, MCF7, and SK-BR-3 as compared to that of the normal breast Hs 578Bst cells (Figure 1(d)). The above results indicated a high expression pattern of GRM8 in breast cancer tissues and cells.

### 3.2. GRM8 Expression Level Is Closely Related to Shorter Overall Survival Time of Patients with Breast Cancer

Then, we explored the clinical value of GRM8 in predicting the overall survival of breast cancer. The Kaplan–Meier curve showed that patients with GRM8 high expression had a lower overall survival rate than that of patients with GRM8 low expression (Figure 1(e)), suggesting that the high expression of GRM8 predicted a poor outcome of patients with breast cancer.

### 3.3. GRM8 Promotes Breast Cancer Cells Transformation to Malignant Phenotype

**Figure 2:**
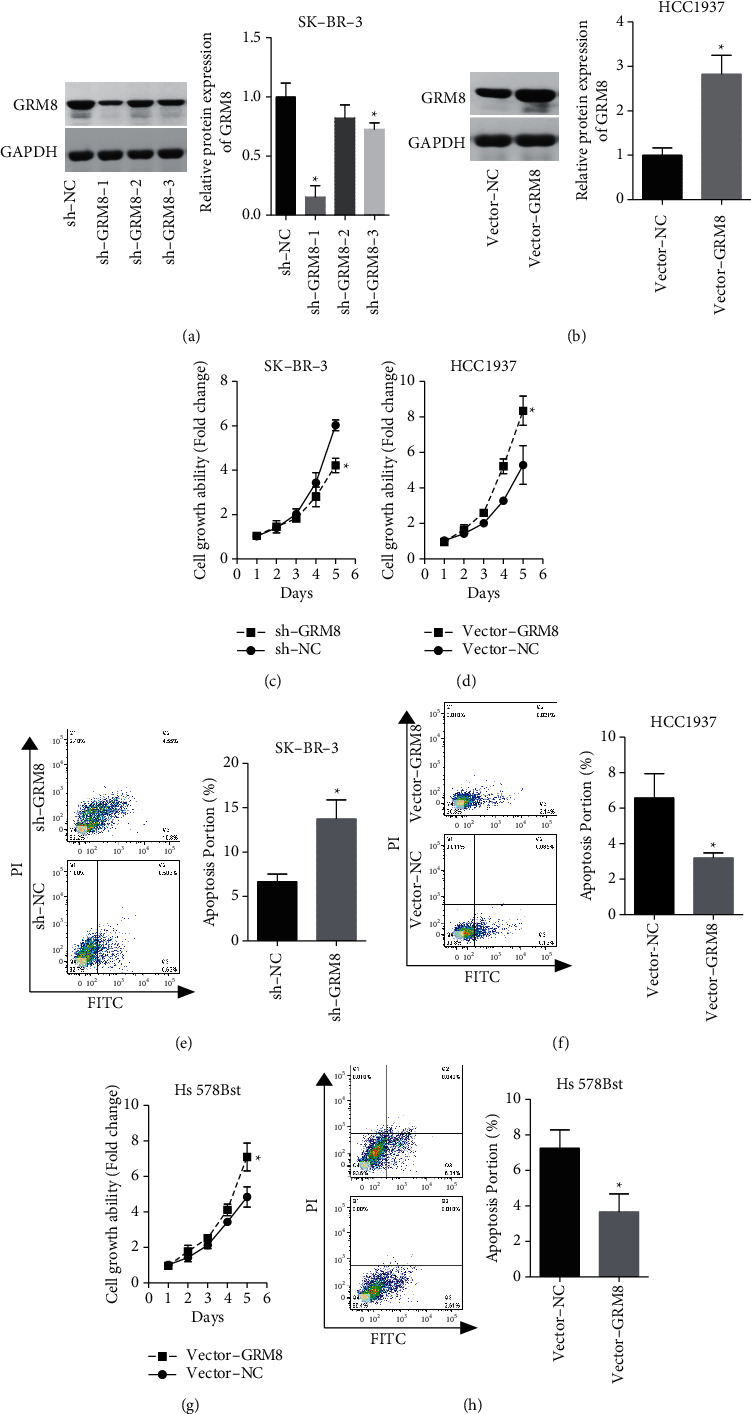
Evaluation GRM8 effects on breast cancer cell growth and apoptosis. (a, b) The expression of GRM8 protein was detected by western blotting assay after SK-BR-3 cells were transected with sh-GRM8/sh-NC or HCC1937 cells were transfected with vector-GRM8/vector-NC. (c, d) CCK-8 assay was used to assess cell growth after SK-BR-3 cells were transected with sh-GRM8/sh-NC or HCC1937 cells were transfected with vector-GRM8/vector-NC. (e, f) Cell apoptosis was detected by flow cytometry assay after SK-BR-3 cells were transected with sh-GRM8/sh-NC or HCC1937 cells were transfected with vector-GRM8/vector-NC. (g, h) CCK-8 and flow cytometry assays were used to detect cell growth and apoptosis in GRM8 overexpressed Hs 578Bst cells (^*∗*^*P* < 0.05).

Then, we explored GRM8 role in breast cancer progression through *in vitro* experiments. As SK-BR-3 showed the highest level of GRM8 among the above breast cell lines, we transfected sh-GRM8 to SK-BR-3 cells to downregulate GRM8 expression. On the contrary, HCC1937 cells were transfected with Vector-GRM8 to overexpress GRM8. The results showed that GRM8 expression was significantly reduced when SK-BR-3 cells were transfected with sh-GRM8-1 and sh-GRM8-2, and sh-GRM8-1 showed the highest knockdown efficiency and was chosen for the following experiments (Figure 2(a)). Vector-GRM8 transfection induced an obvious increase in GRM8 expression in HCC1937 cells (Figure 2(b)). And downregulation of GRM8 significantly inhibited cell viability (Figure 2(c)) and induced apoptosis (Figure 2(e)) in SK-BR-3 cells. Inversely, upregulation of GRM8 significantly promoted cell viability (Figure 2(d)) and inhibited apoptosis (Figure 2(f)) in HCC1937 cells, as well as in normal breast epithelial cell line Hs 578Bst (Figures 2(g) and 2(h)).

**Figure 3:**
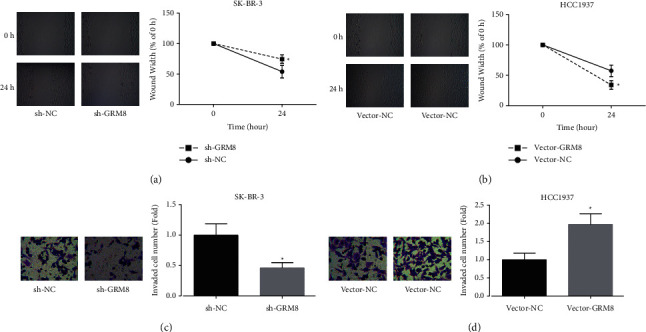
Evaluation GRM8 effects on breast cancer cell migration and invasion. (a, b) Wound healing assay was used to assess cell migration after SK-BR-3 cells were transected with sh-GRM8/sh-NC or HCC1937 cells were transfected with vector-GRM8/vector-NC. (c, d) The effects of GRM8 on invasion of SK-BR-3 and HCC1937 cells were detected by transwell chambers (^*∗*^*P* < 0.05).

In addition, we assessed GRM8 role in regulating breast cancer cell migration and invasion. Knockdown of GRM8 obviously inhibited SK-BR-3 cell migration (Figure 3(a)) and invasion (Figure 3(c)), whereas upregulation of GRM8 in HCC1937 cells caused notable increases in cell migration (Figure 3(b)) and invasion (Figure 3(d)). These results illustrated that GRM8 served as an oncogene in breast cancer.

### 3.4. miR-33a-5p Negatively Modulates GRM8 Expression in Breast Cancer

**Figure 4:**
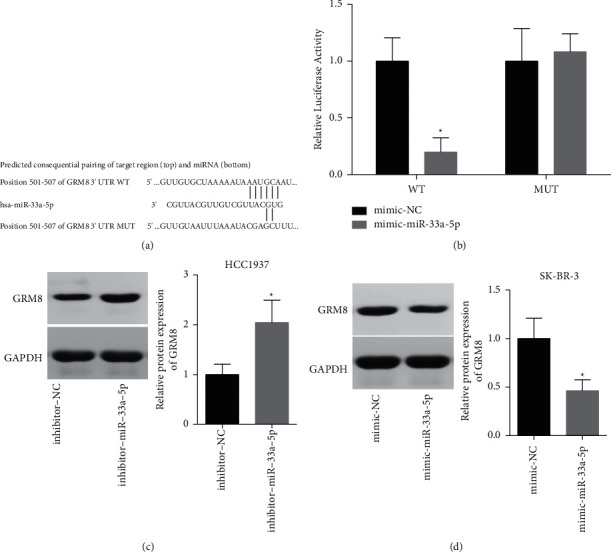
miR-33a-5p negatively regulated GRM8 expression. (a) The binding sites between miR-33a-5p and GRM8. (b, c) Luciferase gene reporter assay was used to assess the relationship between miR-33a-5p and GRM8. (d, e) The expression of GRM8 was detected by western blotting assay after miR-33a-5p was deregulated in SK-BR-3 and HCC1937 cells ( ^*∗*^*P* < 0.05).

Then, we explored the relationship between miR-33a-5p and GRM8 in breast cancer HCC1937 and SK-BR-3 cells.  Figure 4(a) showed the putative binding sites between miR-33a-5p and GRM8. Upregulation of miR-33a-5p with mimic transfection significantly reduced the luciferase activity, whereas this effect was abolished when the binding sites were mutated, as determined by the luciferase gene reporter assay in SK-BR-3 cells (Figure 4(b)). To further confirm the relationship between miR-33a-5p and GRM8, we then performed the western blotting assay. The results showed that miR-33a-5p negatively regulated GRM8 expression in breast cancer HCC1937 and SK-BR-3 cells (Figures 4(c), 4(d)). These results demonstrated that miR-33a-5p negatively modulated GRM8 expression in breast cancer.

### 3.5. miR-33a-5p Inhibits the Progression of Breast Cancer through Downregulating GRM8

**Figure 5:**
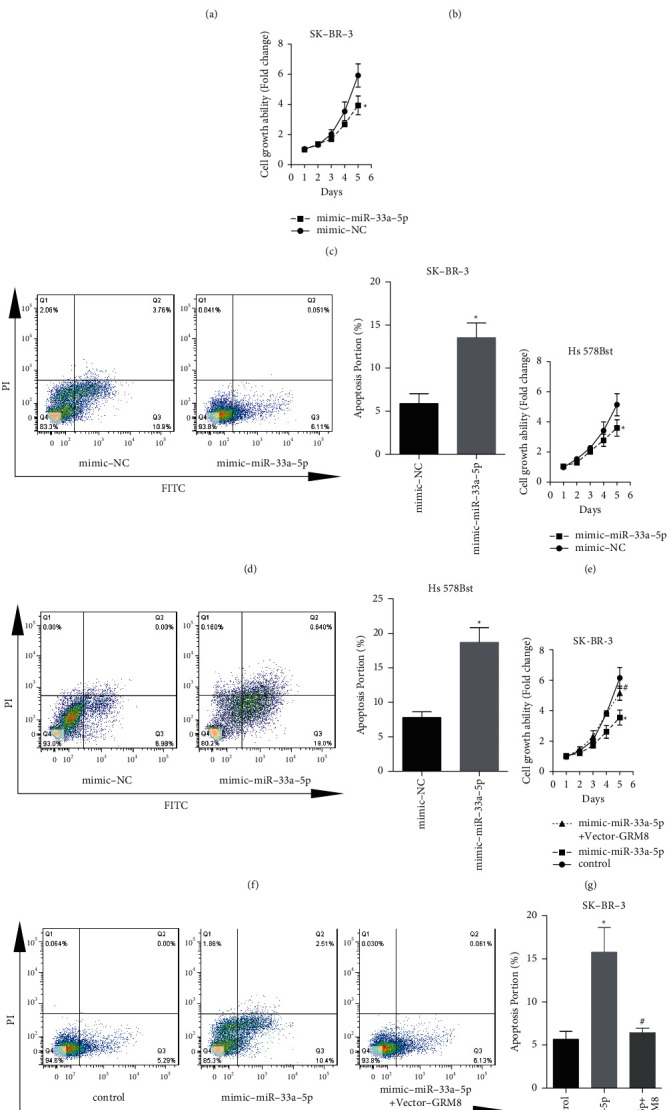
miR-33a-5p inhibited breast cancer cell proliferation and increased cell apoptosis through downregulating GRM8 expression. (a) miR-33a-5p expressions in 80 matched breast cancer tissues and normal tissues were detected by RT-PCR assay. (b) The clinical value of miR-33a-5p in predicting the overall survival rate of breast cancer patients was assessed by the survivorship curve. SK-BR-3 and Hs 578Bst cells were transiently transfected with mimic-miR-33a-5p or the negative controls (mimic-NC); then cell growth was detected by (c, e) CCK-8 assay and cell apoptosis was assessed by (d, f) flow cytometry assay (^*∗*^*P* < 0.05). (g) CCK-8 assay was used to determine cell growth after SK-BR-3 cells were transfected with different vectors. (h) Flow cytometry assay was used to detect cell apoptosis (g, h, ^*∗*^*P* < 0.05, compared with control group; ^#^*P* < 0.05, compared with mimic-miR-33a-5p group).

**Figure 6:**
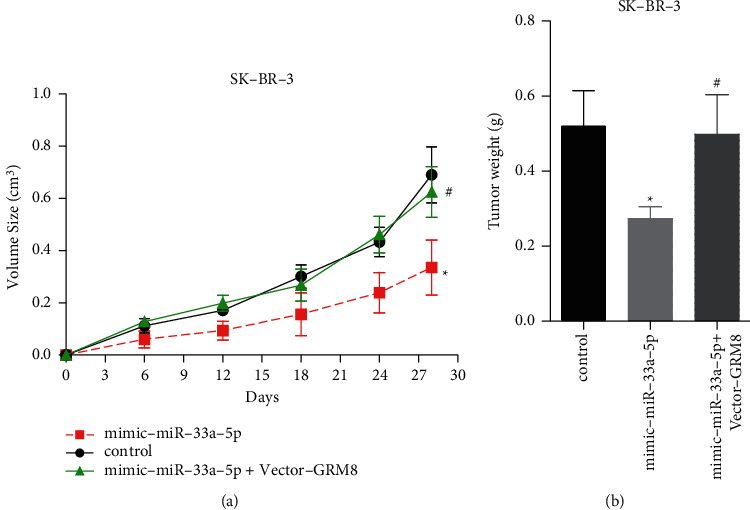
miR-33a-5p inhibited cell tumorigenesis through downregulating GRM8 expression in breast cancer. (a, b) Xenotransplantation experiments were carried out to assess the *in vivo* tumor formation ability after SK-BR-3 cells were stably transfected with NC, mimic-miR-33a-5p, or mimic-miR-33a-5p + vector-GRM8 (^*∗*^*P* < 0.05, compared with control group; ^#^*P* < 0.05, compared with mimic-miR-33a-5p group).

Then, we explored miR-33a-5p/GRM8 axis in the progression of breast cancer. The expression of miR-33a-5p was significantly decreased in breast cancer tissues as compared with the normal tissues (Figure 5(a)), which was significantly associated with lower overall survival rate (Figure 5(b)). Upregulation of miR-33a-5p significantly repressed cell growth and induced apoptosis in SK-BR-3 (Figures 5(c) and 5(d)) and normal breast epithelial cell line Hs 578Bst cells (Figures 5(e) and 5(f)). However, GRM8 overexpression abolished the above roles of miR-33a-5p (Figures 5(g) and 5(h)). In addition, GRM8 upregulation rescued miR-33a-5p-mediated tumor growth inhibition *in vivo* (Figures 6(a) and 6(b)). These results suggested that miR-33a-5p inhibited the progression of breast cancer by targeting GRM8.

## 4. Discussion

Herein, we focused on the role and mechanism of GRM8 in breast cancer progression for the first time, and the results demonstrated that GRM8 was significantly overexpressed in breast cancer, which predicted poor prognosis. In addition, GRM8, under the negative modulation of miR-33a-5p, functioned as an oncogene in the progression of breast cancer.

Up to now, several members of GRMs family have been identified to exert important roles in carcinogenesis. For instance, the high expression of GRM5, a member of group I, was reported to be related to the improved overall survival in human oral squamous cell carcinomas [19]. The antagonist of GRM5 could significantly induce a reduction in cell proliferation of laryngeal cancer RK33 and RK45 cells [20], and GRM5 overexpression promoted melanoma development in transgenic mice [21], indicating that GRM5 functions as an oncogene in laryngeal cancer and melanoma. In addition, works by Speyer et al. [22] revealed that the expression of GRM1, a member of group II, was increased in breast cancer, and knockdown of GRM1 leaded to an obvious inhibition in cell proliferation. Also, Sexton et al. [23] reported that GRM1 was closely associated with the inflammation in triple-negative breast cancer. Moreover, Zhang et al. [24, 25] revealed that the activation of GRM4, a member of group III, could notably promote cell apoptosis and repress cell growth in bladder cancer and glioblastoma cells. GRM8 is another member of group III and maps to chromosome 7q31.3-q32 and consists of 11 exons. The published reports demonstrate that GRM8 exerts different roles in various malignant cancers. Zhang et al. [7] demonstrated that GRM8 significantly enhanced the survival of squamous cell lung carcinoma cells through inhibiting cAMP pathway and activating MAPK pathway, suggesting that GRM8 serves as an oncogene in squamous cell lung carcinoma. On the contrary, Jantas et al. [8] reported that GRM8 overexpression significantly inhibited cell proliferation and enhanced cell chemosensitivity in human neuroblastoma and glioma cells. Li et al. [26] found that the activation of GRM8 induced by 3,4-DCPG treatment increased lung cancer A549 cell apoptosis. These two reports suggest that GRM8 functions as a tumor suppressive gene in human neuroblastoma, glioma, and lung cancer. Similar to GRM8 role in squamous cell lung carcinoma, we found that GRM8 overexpression in breast cancer HCC1937 cells significantly enhanced cell proliferation, migration, and invasion and repressed cell apoptosis, while knockdown of GRM8 in SK-BR-3 cells induced the opposite results, indicating that GRM8 functions as an oncogene in breast cancer. Inevitably, we observed that GRM8 overexpression promoted normal breast cell growth and inhibited apoptosis. However, GRM8 expression level was significantly higher in breast cancer cells than normal breast cells; thus, we think the GRM8 targeting therapy should be designed by higher affinity with breast cancer cells than normal cells in the future.

To explore the molecular mechanism underlying GRM8 in breast cancer progression, we used bioinformatics to search the predicted regulatory miRNAs of GRM8. We found that miR-33a-5p could target GRM8, which was verified by luciferase gene reporter assay. It is well identified that miR-33a-5p serves as a tumor suppressor in several kinds of cancers. For example, Liu et al. [27] demonstrated that miR-33a-5p overexpression significantly suppressed cell proliferation and weakened cell migration and invasiveness in hepatocellular carcinoma through targeting PNMA1. Guo et al. [28] demonstrated that miR-33a-5p was lowly expressed in hepatocellular carcinoma cells, and overexpression of it could obviously inhibit the EMT process and cell invasion ability via binding to Twist1. Moreover, Li et al. [29] clarified that miR-33a-5p overexpression could enhance the sensitivity of lung adenocarcinoma cells to celastrol. miR-33a-5p could be sponged by long noncoding RNA DANCR, leading to glioma progression [30]. Consistently, in the present study, we identified that miR-33a-5p expression was reduced in breast cancer tissues, which was closely associated with lower overall survival. Moreover, we confirmed that miR-33a-5p served as a tumor suppressor in breast cancer through binding to GRM8.

Collectively, the present study discloses that GRM8, negatively regulated by miR-33a-5p, functions as an oncogene in breast cancer progression. Our study indicates the important role that miR-33a-5p/GRM8 axis plays in breast cancer progression, which might be a potential treatment target in breast cancer.

## Figures and Tables

**Table 1:** Primer sequences of RT-PCR.

Gene	Sense (5′-3′)	Antisense (5′-3′)
GAPDH	CACTAGGCGCTCACTGTTCTCTC	GACCAAATCCGTTGACTCCGA
GRM8	GATCAGCCTCTTGCCCTTGT	TGCATAAAGCATGGCCTCCA
miR-33a-5p	GGAGTGCA TTGTAGTTGC	GTGCAGGGTCCGAGGT
U6	CTCGCTTCGGCAGCACA	AACGCTTCACGAATTTGCGT

## Data Availability

The data and materials used to support the findings of this study are included within the published article.
